# The effects of poverty stereotype threat on inhibition ability in individuals from different income‐level families

**DOI:** 10.1002/brb3.1770

**Published:** 2020-10-22

**Authors:** Shanshan Wang, Dong Yang

**Affiliations:** ^1^ Key Laboratory of Cognition and Personality Department of Psychology Ministry of Education Southwest University Chongqing China

**Keywords:** electrophysiology, evoked potentials, income, inhibition, poverty

## Abstract

**Introduction:**

Poverty is characterized by a scarcity of resources and a threat of certain stereotypes. However, the effects of stereotype threat are largely dependent on various factors, both negative and positive. Few psychophysiological studies have studied the effects of poverty stereotype threats on inhibition ability in wealth and impoverished individuals.

**Methods:**

To fill this gap in the literature, this study used the event‐related potential (ERP) technique to explore the brain mechanisms associated with stereotype threat in 135 participants.

**Results:**

Behavioral results showed that the rich group (participants from higher‐income families) had better inhibition ability than the impoverished group (participants from lower‐income families), with significantly shorter reaction time and significantly greater accuracy for poverty‐related stimuli when in the nonthreat condition. Additionally, poverty stereotype threat could improve performance of the impoverished group for poverty‐related stimuli. The electrophysiological results showed significantly larger P3 mean amplitude and significantly longer P3 latency in the rich group than the impoverished group in the nonthreat condition. Although no significant between‐group differences were found in the threat condition, the results show that the effect of poverty stereotype threat varies with different income‐level persons, for both behavioral and P3 data.

**Conclusion:**

These findings suggest that impoverished people have worse inhibition abilities. Further, poverty stereotype threat has different effects on people according to their income level and could help to explain irrational consumption behaviors in people.

## Graphical abstract

This study measured the effect of stereotyping threat on inhibition and attention abilities in impoverished and rich people using behavioral and ERP measures. We believe that our study makes a significant contribution to the literature because it is among the first to use behavioral and electrophysiological approaches to explore the underlying neural mechanisms of inhibitory differences to poverty‐related stimuli.

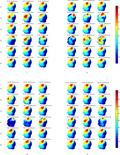

## 1 INTRODUCTION

Poverty is both a simple and complex social phenomenon. It has a great effect on people from different aspects. This study aimed to explore the effects of poverty stereotype threat on inhibitory ability in people from different income‐level families. The purpose of this study was based on the following reasons.

In recent decades, the potential contradiction between the rich and the poor has gradually appeared and several stereotypes of poverty have been established. Inevitably, certain social groups are stigmatized due to their disadvantages, forming the stereotype threat. Stereotype threat refers to the phenomenon that, when negative stereotypes are salient, the stigmatized group performs worse due to fear of confirming these negative stereotypes as characteristic of their ingroup (Steele & Aronson, 1995; Steele, Spencer, & Aronson, 2002). Cozzarelli, Wilkinson, and Tagler (2001) found that there were significantly more negative and few positive stereotypes of the poor than of the middle class. For example, individuals from low‐income families are stereotyped as having inferior intellectual abilities than their peers from high‐income families. When low‐income participants were asked to take intelligence tests, that is, when they were under the income‐based stereotype threat, they performed worse than their high‐income peers (Croizet & Claire, 1998). Another study showed when the participants were all low‐income students, the participants who were informed they took the intelligence test performed worse than the ones who were not informed (Spencer & Castano, 2007). In addition, when low‐income participants were told that poor people generally performed worse than others, they performed worse than when they were not told this information (Harrison, Stevens, Monty, & Coakley, 2006). Taken together, these results indicate the existence of income‐based stereotype threat. Further, poverty stereotype threats have negative effects on individuals’ performance.

Economic scarcity as one of the important factors of poverty makes poor individuals more vulnerable to life pressure and poverty experience. In contrast, low‐income groups are also more likely to experience stress and poverty. Thus, there are interactions between the income‐based stereotype threat and the poverty stereotype threat, and the poverty stereotype threat may even involve broader psychological pressure from living environment or individual personality. This was why, in the poverty stereotype threat operation stage, we involved multiple steps, such as collection of household economic income, presentation of poverty threat materials related to cognition and feedback of participants’ wealth and poverty status, to ensure participants were threatened by poverty stereotypes. In this regard, we assumed that the poverty stereotype threat had different impacts on different income groups, and under the threat, different suppression strategies would be adopted to deal with tasks which could arouse the perception of wealth or poverty.

Few studies have conducted direct research on poverty stereotype threat. Because economic scarcity is one of the basic elements of poverty, we focused on the findings of income‐based stereotype threat. Some researchers have reported that, in people who live in poverty (resource scarcity phenomenon), economic insecurity can reduce personal control (Chou, Parmar, & Galinsky, 2016; Mittal & Griskevicius, 2014). For instance, the participants who were assigned to and informed of the high unemployment rate in their state of residence (i.e., high economic insecurity) showed a greater lack of personal control compared to participants who have no information about their state's unemployment rate (Chou et al., 2016). Self‐control is a key function for human survival and refers to the ability of individuals to adjust their behavior to match both personal values and social expectations. Self‐control can trigger or stop specific behaviors, such as suppressing impulsive behaviors, resisting temptation, delaying gratification, and making future plans. Similarly, Laran and Salerno (2013) manipulated resource scarcity by priming words related to environmental harshness (e.g., struggle, survival), and they found that although individuals knew high‐calorie foods are unhealthy, they would still choose to have more high‐calorie foods, and their intake was far beyond individuals who were not threatened. It even verified that impoverished childhood living environments were also closely correlated to the lack of self‐control under conditions of economic uncertainty (Mittal & Griskevicius, 2014). Therefore, the threat of economic scarcity does have a negative impact on self‐control.

Self‐control is the aspect of inhibitory control that involves control over one's behavior to resist temptations and not act impulsively (Diamond, 2012). Inhibitory control is involved in cognitive inhibition (inhibition of thoughts and memories), selective or focused attention (inhibition of attention), and response inhibition (Diamond, 2012). Therefore, it is inevitable to pay more attention to the ability of inhibitory control. This is also the reason why this study was conducted from the perspective of inhibitory ability.

Additionally, in social practice of poverty alleviation, there is an issue of returning to poverty after the poor get out of the poverty line. Some scholars call it the poverty trap. For this issue, we believe that good self‐control and strong planning ability are not only beneficial for personal financial development but can also help people prevent the recurrence of the poverty trap. However, previous studies have not shown how self‐control was affected by perceived poverty stereotype threats in high‐ and low‐income groups, nor how individual performance changes in different situations (threat vs. nonthreat) when exposed to wealth‐related or poverty‐related stimuli. Therefore, the purpose of this paper was to refine the difference (regarding inhibitory ability) in response to wealth‐related and poverty‐related stimuli in people when they were in or out of the poverty stereotype threat condition.

As we know, when stigmatized individuals experience stereotype threats, they are afraid that they will confirm the negative stereotype to which they belong and, as a result, will engage in efforts to disprove it (Logel, Iserman, Davies, Quinn, & Spencer, 2009; Pennington, Heim, Levy, & Larkin, 2016; Steele & Aronson, 1995). Logel et al. (2009) reported that deliberately suppressing negative thoughts taxes cognitive resources and results in a worse performance and that the effect of stereotype threat alone on performance is partially mediated by pretest thought suppression. Thus, in the present study, we could not be certain that our threat materials would directly cause side effects and considered the possibility that they would also cause improvements. Furthermore, weakness of inhibition ability in impoverished people has not been shown to be a stable characteristic. Thus, this experiment predicted that the individuals from lower‐income families under the poverty stereotype threat would show different resource allocation strategies, compared with their counterparts who were not under the threat. Further, we predicted that there would be differences in resource allocation between lower‐income and higher‐income individuals.

Living in poverty is accompanied by chronic scarcity, which has a series of cognitive consequences that may be beneficial or adaptive in the short term but highly deleterious if experienced chronically (Daminger, Hayes, Barrows, & Wright, 2015; Mullainathan & Shafir, 2013). For example, Shah, Mullainathan, and Shafir (2012) confirmed that scarcity forces individuals to focus their attention on emergencies, showing a highly rational utilization of limited resources. Meanwhile, Anandi Mani, Mullainathan, Shafir, and Zhao (2013) found that scarcity can capture the brain and reduce people's cognitive ability and executive control ability, thus reducing the quality of individual decision making. Therefore, we do not support the conclusion that poverty is completely damaging to individuals, or that it's completely negative when expressed through behavior. Moreover, poverty has obvious relativity due to the fact that self‐assessment is always based on comparisons. For example, middle‐income individuals will feel poor when compared with someone who is richer than them, but they will feel rich when compared with someone who is poorer than them. This may explain one of the underlying reasons for some conflicting results about the effects of poverty. As we know, the impacts of poverty are not as obvious as those of neuron‐trauma, nor directly or obviously reflecting of behavioral performance. Because of this, this paper believed that it may be due to differences in neural activities at the subconscious level, that is, different neural coping strategies are adopted, resulting in insignificant external behavioral differences between poor and rich groups. In other words, differences at the level of neural activity may be more significant than behavioral differences. Thus, on the basis that poverty hinders cognition (Mani et al., 2013; Shah et al., 2012), this paper hypothesized that the differences in neural activity between individuals from high‐income and low‐income families under threat were more obvious than the differences in behavioral performance, and the insignificant differences in external behavior were actually due to different neural coping strategies each participant adopted. Specifically, we expected that individuals from different income‐level families would show different inhibitory capacities on wealth‐related and poverty‐related stimuli, and individuals from lower‐income families may have longer reaction time and lower accuracy, especially under poverty stereotype threat. Further some undetected differences in behavior may show up in neural activity. Therefore, this paper decided to adopt the event‐related potential (ERP) method combined with behavioral outcomes because ERP technology has a high temporal resolution for response of neural activity, and it has been widely utilized in psychological studies to explore relative mental activities.

Previous studies have indicated that the frontal area underlies higher‐level processing; for instance, medial and lateral regions of the prefrontal cortex (PFC), including the anterior cingulate cortex (ACC) and dorsolateral prefrontal cortex, play a prominent role in working memory (Colom et al., 2013; Osaka, Komori, Morishita, & Osaka, 2007) and performance monitoring processes (Osaka et al., 2007; Ullsperger & Cramon, 2001). Previous reviews on stereotype threat (Forbes & Leitner, 2014; Forbes, Schmader, & Allen, 2008) and executive control (Jonkman, 2006; Kanemura, Aihara, Aoki, Araki, & Nakazawa, 2003; Luna & Sweeney, 2004; Sowell et al., 1999) have highlighted the importance of the frontal cortex in monitoring processes and the effects of stereotype threat. Furthermore, ERP studies have reported that the P3 component reflects aspects of information processing that are involved in inhibitory control (Donchin, 1981; Schmitt, Münte, & Kutas, 2000). The P3 component is thought to reflect the allocation of attentional resources (Bauer, Kaplan, & Hesselbrock, 2010; Rosenfeld & Skogsberg, 2006) and inhibitory ability (Baumeister et al., 2014; Nguyen, Moyle, & Fox, 2016). Therefore, according to the aims of this study, we focused on task‐related modulation of the P3 component, which mainly originates from the frontal area.

Furthermore, we referred to and modified the word‐color Stroop paradigm to conduct the research. As we know, researchers often conduct the Stroop task (Hyodo et al., 2012; Tam, 2013) to measure inhibitory ability, especially regarding interference inhibition (Chu, Alderman, Wei, & Chang, 2015; Nigg, 2000; Wöstmann et al., 2013). The Stroop paradigm has been improved and extensively utilized in the cognitive field. With the expansion of research, this classic paradigm has successfully evolved into variants due to exploring different research problems. The emotional Stroop paradigm is one of the variants, which could explore the effect of emotional stimuli on people. This variant utilizes emotional stimuli as priming stimuli and requires the participant to name the color of the target stimuli. Thus, based on the purpose of this study, we modified the Stroop task to conduct our experiments. Based on the purpose of exploring the effects of poverty stereotype threat and poverty‐related or wealth‐related subconscious on people from different income‐level families, we modified the emotional priming stimuli in the emotional Stroop task into poverty‐related and wealth‐related antecedents, which was more suitable for our research.

The antecedents were defined as stimuli presented before the target stimuli to activate the subconscious by semantics of stimuli, based on the experimental purpose. To distinguish from the neutral antecedents, the antecedents with wealth‐related stimuli or poverty‐related stimuli were defined as poverty‐related and wealth‐related antecedents. The purpose of the antecedents was to explore the subconscious influence of wealth and poverty on the subsequent performance when people from different income‐level families were exposed to the threat or nonthreat condition. Meanwhile, the color‐naming words were changed to poverty‐related and wealth‐related stimuli. Similar to the emotional Stroop task, this study instructed participants to name the color of target stimuli and ignore the antecedents and semantic of target stimuli. This study helped to explore the effects of poverty stereotype threat and the poverty‐related and wealth‐related subconscious on inhibitory ability of people from different income‐level families. The changes we made to the emotional Stroop paradigm helped us to achieve the study goal more accurately.

Previous studies have verified the importance of the prefrontal area in higher‐level processing, such as executive control processes (Jonkman, 2006; Kanemura et al., 2003; Luna & Sweeney, 2004; Sowell et al., 1999), task monitoring, and error detection (Carter et al., 1998; Zubicaray, Andrew, Zelaya, Williams, & Dumanoir, 2000). In addition, words were used as stimuli in the current study, which required participants to inhibit semantic processing. Thus, it is not possible to complete the task without any semantic processing. More importantly, stereotype threat has a negative effect on working memory capacity (Schmader, Johns, & Forbes, 2008), and working memory is responsible for the attention to and inhibition of competing information, which are sub‐served by the prefrontal area (Engle, 2001). For general consideration, the left prefrontal and left inferior frontal P3s were analyzed to comprehensively explore our study's questions.

Therefore, we expected to be able to infer social behavior through basic experimental findings. That is, we expected to understand, when people with different economic levels under poverty threat, how their inhibitory ability will affect them, and whether there would be any differences of inhibition of self‐control processing when facing the poverty or wealth labeled goods. This study could help address the following questions: (1) Do stereotype threats affect people from different income‐level families differently?; (2) Under poverty (or income‐based) stereotype threat, do poor individuals significantly change their behaviors after priming of wealth‐related and poverty‐related antecedents?; and (3) Does the inhibition ability of individuals change according to wealth‐related and poverty antecedents under stereotype threats?; and (4) Under poverty stereotype threat, will the neural activities of different groups reveal more significant differences at the subconscious level? And, do the insignificant differences in behavior due to adopting different coping strategies show at the level of neural activity?

## 2 METHODS

### 2.1 Participants

A total of 135 participants were recruited from Southwest University (Mean = 19.45 years old). Participants were allocated to the impoverished group or rich group according to the income standard of poverty households in China as well as trends in arithmetical average incomes and median household incomes. Participants from a family whose per capita income was less than ¥1,100 for rural residents and less than ¥2,250 for urban were defined and placed in the impoverished group (*n* = 68), and the remaining participants were allocated to the rich group (*n* = 67). Due to the random presentation of stereotype materials, 32 participants in the impoverished group and 36 participants in the rich group were presented with poverty stereotype threat materials. Thus, 36 participants of the impoverished group and 31 participants of the rich group were presented with nonstereotype threat materials.

All participants were Chinese native speakers with normal or corrected‐to‐normal vision. The ethics committee of Southwest University approved this research. All participants provided written informed consent.

### 2.2 Materials and procedure

#### 2.2.1 Preparation stage

Participants received simple explanations about the principles of EEG and the experimental procedure before reading and signing a consent form. We collected information about family income status. Participants were told that their family income data would be compared with our database and we would show a family income level feedback of themselves later. However, we did not conduct the comparison, we simply presented a random false feedback to the corresponding group, which was that they belonged to a rich or poor family by comparison. After collecting information, participants were required to read a short report (about 450–500 words) and complete a five‐item questionnaire that was related to the content of the text.

Participants were randomly assigned to a threat or nonthreat condition. Participants in the nonthreat condition read a short scientific report, which briefly described the samples and photographs taken by the Japanese asteroid probe (Hayabusa 2), and were presented with family income level feedback stating that they belonged to a relatively rich family. Participants in the threat condition read the short report, which described the long‐term negative effect of poverty on cognitive processing, and were presented with feedback stating that they belonged to a poverty‐stricken family.

#### 2.2.2 Experimental stage

Thus, according to our aims, we designed a vocabulary list of wealth‐related and poverty‐related words and neutral words by expert assessment. There were three types of antecedents and target stimuli—words associated with wealth, words associated with poverty, and neutral words. Antecedents comprised two Chinese characters in white ink (e.g. poverty‐related: 贫困 (poor); wealth‐related: 富有 [rich]; neutral: 树木 [tree]). Target stimuli were idioms and included four Chinese characters (e.g., poverty‐related: 贫困潦倒 [down and out]; wealth‐related: 富可敌国 [richer than a king]; neutral: 山清水秀 [picturesque scenery]). The thirty experts who participated in the evaluation were graduate students and professors of psychology majors with rich experience of research. The correlation of expression of poverty‐related and wealth‐related words and corresponding meaning of “poverty” or “wealth,” the familiarity of neutral words, and the utilization rate of all words in the vocabulary list were assessed by a nine‐point Likert scoring questionnaire. The results were analyzed according to the kinds of words (poverty‐related, wealth‐related, and neutral) and the types of stimuli (antecedents with two Chinese characters and target stimuli with four Chinese characters). The mean values of correlation and familiarity of each kind of word were calculated with high assessment scores (range from 7.44 to 8.47), and the assessment scores of utilization rate of words in the vocabulary list were from 4.32 to 6.67. The whole evaluation process was in strict accordance with the operation requirements of psychological questionnaire measurement, and we ensured that there was no artificial or experimental interference of peripheral persons.

Participants were required to suppress semantic processing and to focus only on the colors of words. Given that we wanted to study about question 2 we listed above, antecedents, playing as priming stimuli, occurred before the target stimuli to arouse awareness of a wealth/poverty and explore whether this priming would affect the inhibition ability.

A white priming stimulus appeared for 250 ms after the presentation of a 500‐ms fixation cross. Then, a red or blue target stimulus replaced the antecedents on the screen. Participants were required to judge, within 1,500 ms, whether the stimulus was red (by pressing “F”) or blue (by pressing “J”). The intertrial interval was 500 ms, during which a blank screen was presented. There were three types of antecedents and target stimuli—words associated with wealth, words associated with poverty, and neutral words. Antecedents comprised two Chinese characters in white ink. Participants completed a total of 540 trials across three repeated blocks that were presented in a random order. Five practice trials were completed before the formal experiment. All stimuli appeared on a 19‐inch color display with 85 Hz refresh rates, 0.1° spatial resolution, a 1,024 by 768‐pixel resolution, and a fixed viewing distance of approximately 60 cm.

### 2.3 Check manipulation

To check whether participants believed in the false feedback, especially the participants whose household income was far from the threshold, we conducted two open questionnaire surveys. The first questionnaire was a self‐assessment to all participants, after the preparation stage was over and before the formal experiment began, and it was a two‐question questionnaire with the requirement that the participant had to provide at least five answers to each question. The second questionnaire was a survey regarding stereotypes of impoverished people to participants under the threat condition after all experiments were over and before the participants left. The role of the second questionnaire was utilized as supporting proof for the first questionnaire to prevent participants from answering questions without substituting into the prerequisites, in other words, to prevent the individual from not believing the feedback (they were belonged to a relatively rich/poverty family) when they filled out the first questionnaire. To avoid the content of the questionnaire influencing the participants, there were two versions of the first questionnaire survey. For the nonthreatened group, the questions were as follows: “When you compare yourself with high‐tech scientists, what would you say are your strengths?” and “What would you say are your weaknesses?”. For the threatened group, the questions were as follows: “When you compare yourself with a very wealthy man, what would you say are your strengths?” and “What would you say are your weaknesses?”.

Experimenters screened and categorized the key words form the participants’ answers. We analyzed the frequency of each type of word rigorously by using SPSS (v. 22.0). The results revealed that the implementation of poverty stereotype threat was successful in both groups. Specifically, in the threat condition, the participants showed significant feelings of inferiority, poverty, and anxiety, and the word frequency of inferiority was significantly higher than the word frequency in their answers about stereotypes of poverty, indicating that there was indeed a threat effect. The significantly higher frequency of these keywords in the rich group of the threat condition and significantly lower frequency of the keywords in the impoverished group of the nonthreat condition also revealed that they were supposed to believe false feedback, which was contrary to their household income level.

### 2.4 EEG recordings and data reduction

Electroencephalography (EEG) data were recorded continuously from 64 scalp electrodes at a sampling rate of 500 Hz. The references were the left and right mastoids and a ground electrode at the medial frontal site. EEGs and electrooculogram (EOG) were amplified using a 0.05–100 Hz bandpass. All electrode impedance was kept below 5 kΩ. Offline, data were re‐referenced to the average of the left and right mastoids, and a bandpass filter of 0.1–40 Hz was applied. Trials with a horizontal EOG voltage exceeding ±30 μV and epochs in which the amplitude exceeded ±80 μV were rejected. EEG data were segmented into 800‐ms epochs, which included a 200 ms prestimulus baseline recording. Additionally, an independent component analysis was used to remove ocular artifacts (blinks and saccades), and artifact rejection was performed to exclude the effects of muscle or recording artifacts and excessive noise.

### 2.5 Data analysis

#### 2.5.1 Behavioral analysis

A repeated‐measures analyses of variance (ANOVA) was employed to assess reaction time and accuracy according to the following behavioral parameters: condition (threat vs. nonthreat) × group (impoverished vs. rich) × antecedents (poverty vs. wealth vs. neutral) × target stimulus (poverty vs. wealth vs. neutral).

#### 2.5.2 ERP waveform analysis

The ERP analysis was conducted with left inferior frontal sites (F7, F5, FT7) and left prefrontal sites (AF3, F3, F1) of the P3, where the components of interest were maximal. Mean P3 amplitudes and latencies were measured in the 300–500 ms latency window after stimulus onset. ERP components were measured relative to the 200‐ms prestimulus baseline. A repeated‐measures ANOVA was conducted to analyze condition (threat vs. nonthreat) × group (impoverished vs. rich) × antecedents (poverty vs. wealth vs. neutral) × target stimulus (poverty vs. wealth vs. neutral) × accuracy (correct vs. wrong) × electrodes effects.

The threshold for significance was set at *p* < .05, adjusted by sphericity violations and the Greenhouse–Geisser correction. When significant interactions were found, these were analyzed using the Bonferroni correction. All analyses were performed using SPSS (v 22.0).

## 3 RESULTS

### 3.1 Behavioral data

There was a significant main effect of target stimulus (*F*(2, 130) = 5,691.91, *p* < .001, ŋ_p_^2^ = 0.989) and a significant interaction effect of target stimulus × group × condition (*F*(2, 130) = 5.38, *p* = .006, ŋ_p_^2^ = 0.076) on accuracy. Post hoc analysis of the main effect revealed a significantly greater accuracy in response to poverty and wealth target stimuli than to neutral target stimuli (poverty target stimuli: *M* ± *SE* = 0.94 ± 0.01; wealth target stimuli: *M* ± *SE* = 0.93 ± 0.01; neutral target stimuli: *M* ± *SE* = 0.49 ± 0.00). Post hoc analysis of the significant interaction effect revealed a significantly greater accuracy in response to poverty and wealth target stimuli than to neutral stimuli in both groups, independent of condition (all *p's* < .001). In the threat condition, accuracy in response to poverty target stimuli was significantly greater than wealth target stimuli in the impoverished group (*p* < .001). For poverty target stimuli, we found that accuracy of the rich group was significantly greater than that of the impoverished group in the nonthreat condition (*F*(1, 131) = 6.66, *p* = .011, ŋ_p_^2^ = 0.048). Within the impoverished group, response accuracy to poverty target stimuli was significantly greater in the threat condition than in the nonthreat condition (*F*(1, 131) = 4.47, *p* = .036, ŋ_p_^2^ = 0.033). Details of associated mean and standard error of significant interaction effect we found were presented in the Table 1.

**TABLE 1:** Mean and standard error of accuracy of the interaction effect of target stimulus × group × condition in both groups in each condition

Target stimuli	Group	Condition	Mean	*SE*
Poverty‐related	IG	TC	0.95	0.01
		NC	0.92	0.01
	RG	TC	0.94	0.01
		NC	0.95	0.01
Wealth‐related	IG	TC	0.93	0.01
		NC	0.92	0.01
	RG	TC	0.94	0.01
		NC	0.94	0.01
Neutral	IG	TC	0.50	0.003
		NC	0.49	0.003
	RG	TC	0.49	0.003
		NC	0.49	0.003

Abbreviations: IG, participants from lower‐income families; NC, did not in the poverty stereotype threat condition; RG, participants from higher‐income families; TC, in the poverty stereotype threat condition.

We found a significant main effect of target stimulus (*F*(2, 130) = 117.36, *p* < .001, ŋ_p_^2^ = 0.644) and significant interaction effects for target stimulus × group × condition (*F*(2, 130) = 3.59, *p* = .030, ŋ_p_^2^ = 0.052) and antecedents × target stimulus × group (*F*(4, 128) = 1.22, *p* = .029, ŋ_p_^2^ = 0.080) on reaction times. Post hoc analysis of the significant main effect revealed that reaction times to poverty and wealth target stimuli were significantly longer than that for neutral target stimuli (poverty target stimuli: *M* ± *SE* = 477.94 ± 8.32; wealth target stimuli: *M* ± *SE* = 475.56 ± 8.20; neutral target stimuli: *M* ± *SE* = 451.18 ± 8.37). Post hoc analysis of significant interaction effects indicated that reaction times to poverty and wealth target stimuli were significantly longer than that for neutral target stimuli in both groups, across all conditions, and regardless of the antecedents used (all *p's* < .001). In addition, the impoverished group had significantly longer reaction times than the rich group for all kinds of target stimuli in the nonthreat condition (poverty target stimuli: *F*(1, 131) = 6.23, *p* = .014, ŋ_p_^2^ = 0.045; wealth target stimuli: *F*(1, 131) = 4.44, *p* = .037, ŋ_p_^2^ = 0.033; neutral target stimuli: *F*(1, 131) = 5.47, *p* = .021, ŋ_p_^2^ = 0.040). All details of associated mean and standard error of significant interaction effects are presented in Tables 2 and 3.

**TABLE 2:** Mean and standard error of reaction time of interaction effect of target stimulus × group × condition in each condition

Target stimuli	Group	Condition	Mean	*SE*
Poverty‐related	IG	TC	479.06	17.05
		NC	503.39	16.07
	RG	TC	484.90	16.07
		NC	444.42	17.32
Wealth‐related	IG	TC	474.92	16.81
		NC	498.82	15.85
	RG	TC	478.75	15.85
		NC	449.74	17.08
Neutral	IG	TC	445.88	17.16
		NC	477.42	16.18
	RG	TC	459.63	16.18
		NC	421.79	17.44

Abbreviations: IG, participants from lower‐income families; NC, did not in the poverty stereotype threat condition; RG, participants from higher‐income families; TC, in the poverty stereotype threat condition.

**TABLE 3:** Mean and standard error of reaction time of interaction effect of antecedents × target stimulus × group in each condition

Target stimuli	Group	Condition	Mean	*SE*
Poverty‐related	IG	TC	493.21	12.42
		NC	461.37	12.52
	RG	TC	489.64	12.42
		NC	467.85	12.52
Wealth‐related	IG	TC	460.20	12.61
		NC	439.75	12.72
	RG	TC	486.24	11.24
		NC	468.56	11.33
Neutral	IG	TC	486.30	11.20
		NC	461.99	11.30
	RG	TC	464.05	11.96
		NC	439.09	12.06

Abbreviations: IG, participants from lower‐income families; NC, did not in the poverty stereotype threat condition; RG, participants from higher‐income families; TC, in the poverty stereotype threat condition.

### 3.2 ERP data

Regarding the left prefrontal P3, there was a significant main effect of group (*F*(1, 131) = 4.82, *p* = .030, ŋ_p_^2^ = 0.035) on P3 amplitude and a significant interaction effect for antecedents × group × condition (*F*(2, 130) = 3.61, *p* = .030, ŋ_p_^2^ = 0.053) on P3 latency. Regarding the amplitude of the left inferior frontal P3, there was a significant antecedent × group ×condition interaction effect (*F*(2, 130) = 3.53, *p* = .032, ŋ_p_^2^ = 0.052). Regarding the latency of the left inferior frontal P3, there were significant antecedents × group × condition interaction (*F*(2, 130) = 3.77, *p* = .026, ŋ_p_^2^ = 0.055) and antecedents × target stimulus × accuracy × group interaction (*F*(4, 128) = 2.90, *p* = .024, ŋ_p_^2^ = 0.083) effects.

Post hoc analysis for the main effects revealed that left prefrontal P3 amplitude was significantly larger in the rich group than in the impoverished group (impoverished group: *M* ± *SE* = 3.63 ± 0.39; rich group: *M* ± *SE* = 4.84 ± 0.39). The post hoc tests for the significant interaction effects revealed the following simple effects.

In the nonthreat condition: (a) When antecedents were wealthy words, it showed significantly longer latency and larger amplitude in the rich group than in the impoverished group, reflecting the left prefrontal P3 latency (*F*(1, 131) = 8.82, *p* = .004, ŋ_p_^2^ = 0.063), the left inferior frontal P3 latency (*F*(1, 131) = 6.22, *p* = .014, ŋ_p_^2^ = 0.045), and the left inferior frontal P3 amplitude (*F*(1, 131) = 4.98, *p* = .027, ŋ_p_^2^ = 0.037); (b) When antecedents were poverty words, the left inferior frontal P3 amplitude was significantly larger in the rich group than in the impoverished group (*F*(1, 131) = 5.05, *p* = .026, ŋ_p_^2^ = 0.037); (c) The left prefrontal P3 latency was significantly longer in response to wealth and neutral antecedents than in response to poverty antecedents in the rich group (*F*(2, 130) = 6.90, *p* = .001, ŋ_p_^2^ = 0.096).

In the threat condition: (a) The rich group showed significantly longer latency and larger amplitude in response to neutral antecedents than in response to poverty and wealth antecedents in the rich group, reflected in the left prefrontal P3 latency (*F*(2, 130) = 6.48, *p* = .002, ŋ_p_^2^ = 0.091) and the left inferior frontal P3 amplitude (*F*(2, 130) = 5.31, *p* = .006, ŋ_p_^2^ = 0.075). (b) In the rich group, the left prefrontal P3 latency in the threat condition was significantly longer than in the nonthreat condition (*F*(2, 130) = 4.36, *p* = .039, ŋ_p_^2^ = 0.032). (c) In the impoverished group, the left inferior frontal P3 amplitude was significantly larger in response to wealthy antecedents than to poverty antecedents (*F*(2, 130) = 3.46, *p* = .034, ŋ_p_^2^ = 0.051).

**FIGURE 1:**
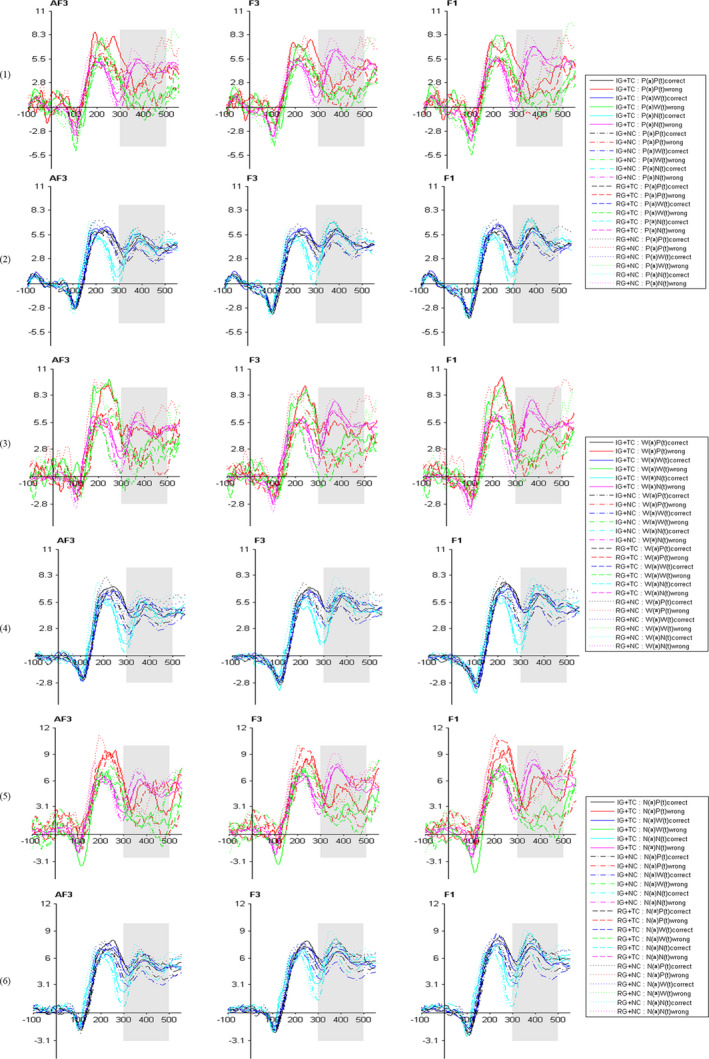
Grand‐average ERP waveforms at AF3, F3, and F1 electrode sites for all conditions. IG, Impoverished group; RG, Rich Group; TC, Threat Condition; NC, Nonthreat Condition; P, Poverty stimuli; W, Wealth stimuli; N, Neutral stimuli; (a) = antecedents; (t) = target stimuli; (1) = trails with poverty antecedents and respond correctly; (2) = trails with poverty antecedents and respond incorrectly; (3) = trails with wealth antecedents and respond correctly; (4) = trails with wealth antecedents and respond incorrectly; (5) = trails with neutral antecedents and respond correctly; (6) = trails with neutral antecedents and respond incorrectly

**FIGURE 2:**
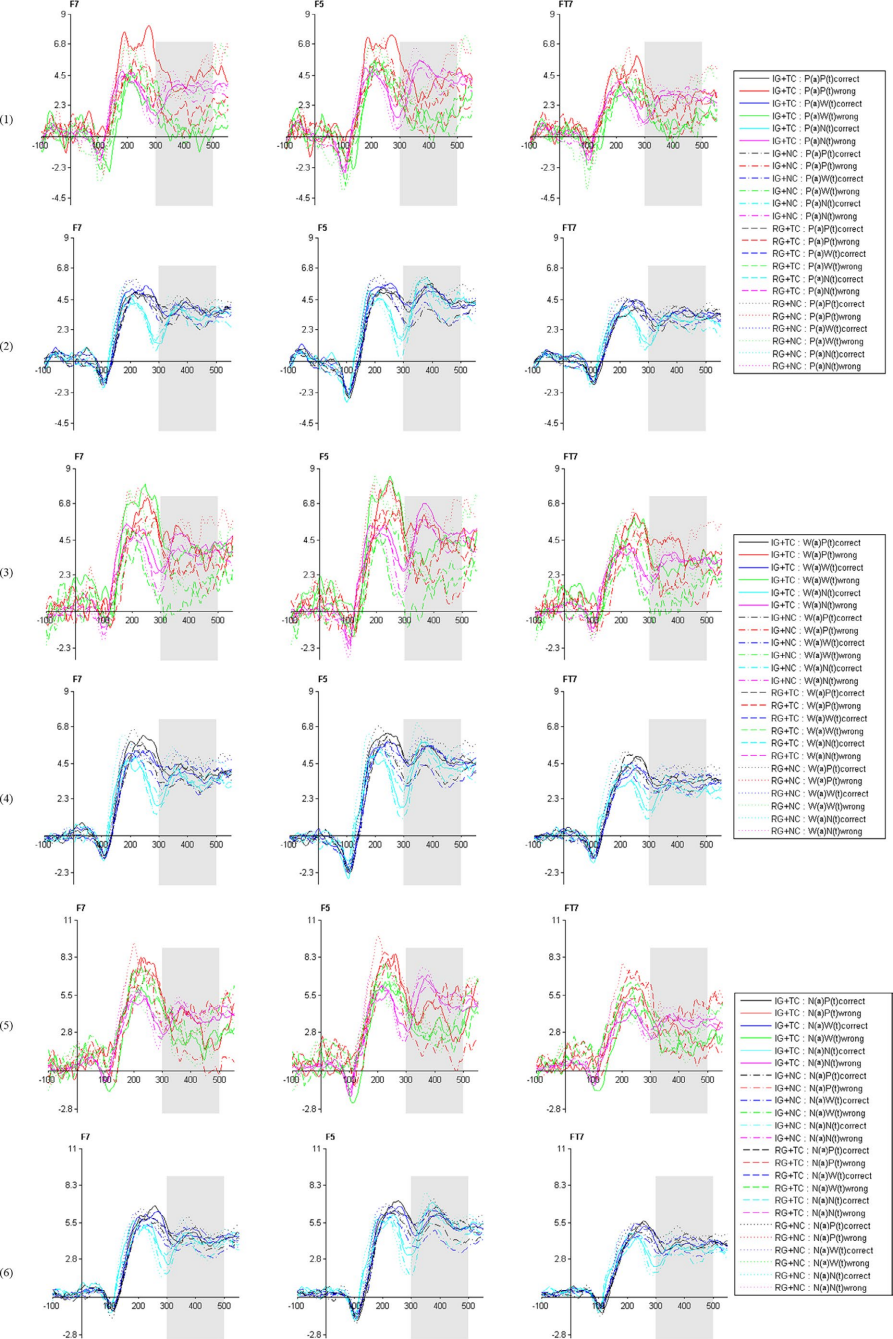
Grand‐average ERP waveforms at F7, F5, and FT7 electrode sites for all conditions. IG = Impoverished group; RG = Rich Group; TC = Threat Condition; NC = Nonthreat Condition; P = Poverty stimuli; W = Wealth stimuli; N = Neutral stimuli; (a) = antecedents; (t) = target stimuli; (1) = trails with poverty antecedents and respond correctly; (2) = trails with poverty antecedents and respond incorrectly; (3) = trails with wealth antecedents and respond correctly; (4) = trails with wealth antecedents and respond incorrectly; (5) = trails with neutral antecedents and respond correctly; (6) = trails with neutral antecedents and respond incorrectly

**FIGURE 3:**
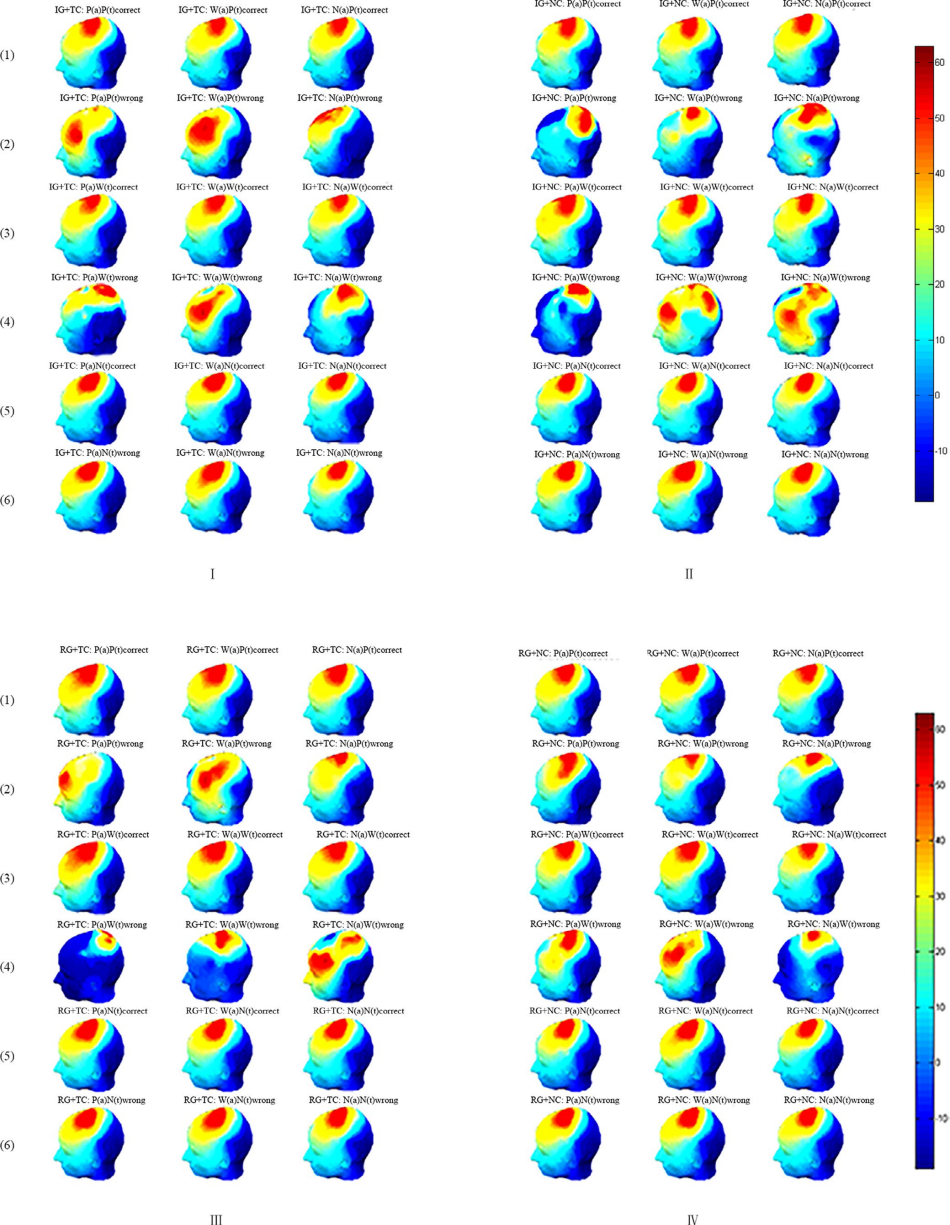
Topographical maps in 400 ms in different conditions. IG, Impoverished group; RG, Rich Group; TC, Threat Condition; NC, Nonthreat Condition; P, Poverty stimuli; W, Wealth stimuli; N, Neutral stimuli; (a) = antecedents; (t) = target stimuli; I = impoverished group in threat condition; II = impoverished group in nonthreat condition; III = rich group in threat condition; IV = rich group in nonthreat condition; (1) = trails with poverty antecedents and respond correctly; (2) = trails with poverty antecedents and respond incorrectly; (3) = trails with wealth antecedents and respond correctly; (4) = trails with wealth antecedents and respond incorrectly; (5) = trails with neutral antecedents and respond correctly; (6) = trails with neutral antecedents and respond incorrectly

Figure 1 shows the specific differences of the left prefrontal P3, Figure 2 presents the differences of the left inferior frontal P3 in all conditions, and Figure 3 demonstrates specific differences in the scalp map.

## 4 DISCUSSION

The main objective of the current study was to explore poverty stereotype threat on inhibitory differences to wealth‐related and poverty‐related stimuli in people from different income‐level families. To the best of our knowledge, this is the first study to use behavioral and electrophysiological approaches to explore underlying neural mechanisms of such differences. The results revealed that poverty stereotype threat results in improvements in response accuracy for poverty‐related stimuli and significantly greater effect in lower‐income individuals. Moreover, it revealed significant group differences in reaction time, accuracy, P3 mean amplitude, and latency.

Specifically, behavioral results showed that poverty stereotype threat can enhance the inhibition ability of people from lower‐income families, and the improvement of inhibition ability is greatest for poverty‐related stimuli with significantly improved accuracy. This is not the case in individuals from higher income‐level families. Moreover, when there were no poverty stereotype threats, individuals from higher income‐level families experienced a significantly shorter reaction time to all kinds of stimuli and significantly greater accuracy for poverty‐related stimuli than individuals from lower income‐level families. These behavioral findings suggest that people from higher‐income families have better ability to control motor response inhibition, especially for poverty‐related stimuli. In addition, poverty stereotype threat has the effect to reduce the group discrepancy of inhibition ability, and it will cause people from lower‐income families to be more sensitive to poverty‐related stimuli. Steele and Aronson (1995) reported that when individuals were threatened by a negative stereotype, either about themselves or about the group they belonged to, they worried about verifying those negative stereotypes and subsequently made more of an effort to disprove it (Logel et al., 2009; Pennington et al., 2016; Steele & Aronson, 1995), to perform better (O'Brien & Crandall, 2003). Therefore, significant group discrepancies disappeared, perhaps because persons from lower‐income families make an effort to improve their performance and to disprove the negative stereotypes about themselves after realizing the presence of the poverty stereotype. In other words, poverty stereotype threat has an improvement effect on inhibition ability, especially for poverty‐related stimuli, in persons from lower‐income families.

Activation of the stereotype is affected by many factors and can be consciously inhibited and controlled by individuals rather than being completely automated (Blair, 2002). Stereotype threat is primarily and negatively affected by stereotype, which does not appear all the time and requires external stimuli to induce it. Additionally, its effects may be different between distinct groups and situations (Beilock, Rydell, & Mcconnell, 2007; O'Brien & Crandall, 2003; Rydell & Boucher, 2010). Given this view, we tried to explore whether the absence of any significant differences in the stereotype threat condition was due to different effects. Combined with the results of insignificant group differences in poverty stereotype threat condition, we found the opposite effect of poverty stereotype threat on both groups. This opposite effect was demonstrated through reaction time, accuracy, and ERPs. Using reaction time as an example, compared with the nonthreat condition, reaction time was shortened in the impoverished group and was prolonged in the rich group when in the poverty stereotype threat condition. Thus, combined with O'Brien and Crandall's findings (2003), we believed that absence of any significant differences in the threat condition was because of the opposite effects of poverty stereotype threat on the impoverished and rich groups. Therefore, we support that the effect of poverty stereotype threat varies with persons from different income‐level families. However, no significant differences in the threat condition were ultimately found, yet the exact opposite effects of the poverty stereotype threat on groups cannot be pointed out directly. It only can suggest the trending of the opposite effect of stereotype threat on different income‐level crowds.

The results of ERP showed significant differences regarding antecedents between the groups. It comprehensively revealed significantly larger P3s mean amplitude and significantly longer P3 latency after wealth‐related antecedents in higher‐income people than lower‐income people, when there was no poverty stereotype threat. Amplitude of P3 component can reflect attention resource allocation during information processing, and latency is thought to be an index of stimulus classification and speed evaluation, independent of response selection and action. A greater P3 amplitude has also been associated with an upregulation of inhibitory control (Drollette et al., 2014; Hillman et al., 2009; Pontifex, Saliba, Raine, Picchietti, & Hillman, 2013), and a shorter latency indicates a faster processing speed (Duncan‐Johnson, 1981; Verleger, 1997). Our findings suggest people from higher income‐level families have better inhibition ability, with more attention resources allocated, for poverty‐related and wealth‐related stimuli than peers from lower income‐level families in the nonthreat condition, within a trade‐off with latency. Analogous to the behavioral results, we did not reveal any group differences in the poverty stereotype threat condition. To this, we keep the same view as above, that is, poverty stereotype threat will promote the elimination of the significant group difference in inhibition ability and has the opposite effect on people from different income‐level families. However, we cannot be sure whether poverty stereotype threat has a positive or negative impact on individuals from lower‐income or higher‐income families.

In this study, participants were affected by antecedents as well, except for responding to the target stimuli when they were either in the threat condition or in the nonthreat condition. Antecedents were wealth‐related, poverty‐related, and neutral words and differed from words of the target stimuli. The antecedents we utilized were more concise and clearer, and the word frequency shown in the Chinese corpus is higher than the target words used in the current study. Poverty stereotype was threatened to participants, and we assumed that the antecedents resulted in a wealth‐related or poverty‐related awareness by concepts of wealth‐related and poverty‐related words. We aimed to explore whether poverty stereotype threat had a different effect on people from distinct income‐level families and to explain the social behaviors in lower‐income individuals as much as possible. Given the results of previous income‐based stereotype threat studies, when participants were under income‐based stereotype threat, low‐income participants performed worse in the intelligence tests than high‐income counterparts (Croizet & Claire, 1998); and when low‐income participants were under income‐based stereotype threat, participants who were informed to take intelligence tests performed worse than those who were not informed (Spencer & Castano, 2007). Therefore, antecedents were within‐participants factors to arouse different awareness, and poverty stereotype threat was a between‐group factor in the current study. Interestingly, although the behavioral results did not reveal the effect of antecedents on inhibition ability, the ERP results did. Specifically, poverty stereotype threat had a more significant effect on processing speed of wealth‐related antecedents in persons from higher income‐level families and lead to significant differences of wealth‐related and poverty‐related antecedents in the nonthreat condition in neural activities to disappear. However, significant differences of attention allocation emerged on the wealth‐related and poverty‐related antecedents in individuals from lower‐income families due to the effect of poverty stereotype threat. In addition, it shows that poverty stereotype threat will reduce group differences. In summary, poverty stereotype threat has different effects on people from different income‐level families, and the finding of different neural activities could help to clarify the differences found in behavioral outcomes in the nonthreat condition and the disappearance of the behavioral differences of groups in the threat condition. That is, when under the nonthreat condition, it is due to more attention allocation by people from higher income‐level families than their peers that reflects significant faster action time with less reaction time. However, the threat condition promotes people from higher‐income families to reduce the differences of processing speed and resource allocation between wealth‐related and poverty‐related stimuli and promotes people from lower‐income families to increase differences of resource allocation of wealth‐related and poverty‐related stimuli. Thus, it explains the disappearance of behavioral differences between groups from the perspective of neural activities. Further, the neural outcomes also helped explain the automatic processing of people from lower‐income families under the threat condition, showing the significant differences of accuracy between wealth‐related and poverty‐related stimuli in people from lower‐income families.

Poverty is accompanied by distinct kinds of resource scarcity, which can lead to greater categorization and stereotyping of individuals (Krosch & Amodio, 2014; Rodeheffer, Hill, & Lord, 2012) and can deplete finite self‐regulatory resources (Mani et al., 2013; Vohs, 2013). For example, Cannon, Goldsmith, and Roux (2019) reported that, in consumers who were financially instable, resource scarcity threatened consumers’ sense of personal control when they faced the problem of reducing the resource discrepancy in resource levels. Some people, who suffer the economic scarcity, engage in the unnecessary consumption behaviors to reduce the sense of financial security, even if the money scarcity will become worse after doing this (Cannon et al., 2019). Poor inhibitory control is highly associated with social, behavioral, and cognitive problems (Ozonoff & Jensen, 1999; Schultz, Evans, & Wolff, 1999), including irrational consumption behaviors. The present findings revealed that the inhibitory ability of people from lower income‐level families did worse than people from higher income‐level families, and poverty stereotype threat could reduce the group differences especially for poverty‐related stimuli, even improving their performance of inhibition. Thus, we may help lower‐income consumers who make irrational consumption decisions to eliminate the sense of scarcity insecurity and to make more reasonable plans with better inhibition ability under poverty stereotype threat to a certain extent. Moreover, we did not find the significant effect of arousing poverty‐related or wealth‐related awareness on impoverished individuals’ inhibition ability. Therefore, it is not necessary to inform impoverished people who have irrational consumption behaviors of their wealth state, when they were under poverty stereotype threat. In conclusion, the effect of poverty stereotype threat is not entirely negative for impoverished people, and it can even improve impoverished people's inhibitory ability to some extent.

## CONFLICTS OF INTEREST

The authors have no conflicts of interest to declare.

## AUTHOR CONTRIBUTION

All authors contributed to conception and design of the study. Shanshan Wang organized the database and performed the statistical analysis. Shanshan Wang and Dong Yang wrote the first draft of the manuscript. All authors contributed to manuscript revision, read, and approved the submitted version and agree to be accountable for the content of the work.

### Peer Review

The peer review history for this article is available at https://publons.com/publon/10.1002/brb3.1770.

## Data Availability

All relevant data are available via Figshare at DOI: https://doi.org/10.6084/m9.figshare.12044478
